# Raltegravir Attenuates Experimental Pulmonary Fibrosis *In Vitro* and *In Vivo*

**DOI:** 10.3389/fphar.2019.00903

**Published:** 2019-08-20

**Authors:** Xue Zhang, Haidi Huang, Guanghua Zhang, Defang Li, Hongbo Wang, Wanglin Jiang

**Affiliations:** ^1^School of Pharmacy, Binzhou Medical University, Yantai, China; ^2^School of Pharmacy, Yantai University, Yantai, China

**Keywords:** Raltegravir, pulmonary fibrosis, NLRP3 inflammation, NLRP3 inhibitor, HMGB1/TLR4/NF-κB

## Abstract

Raltegravir, an inhibitor of human immunodeficiency virus-1 (HIV-1) integrase, has been used to treat HIV/acquired immunodeficiency syndrome; however, its therapeutic effects on pulmonary fibrosis have not been investigated. In this study, the *in vitro* effects of raltegravir (RAV) on transforming growth factor beta 1 (TGF-β1)-induced pulmonary fibrosis on L929 mouse fibroblasts were investigated. In addition, the effects of RAV on an *in vivo* pulmonary fibrosis model induced by intratracheal instillation of bleomycin were investigated. The proliferation of L929 cells was inhibited after RAV treatment. Meanwhile, the *in vitro* and *in vivo* protein expression of nucleotide-binding oligomerization domain-like receptor 3 (NLRP3), high-mobility group box 1 (HMGB1), toll-like receptor 4 (TLR4), prolyl hydroxylase domain protein 2, phosphorylated nuclear factor-κB (p-NF-κB), hypoxia-inducible factor-1α (HIF-1α), collagens I and III was reduced relative to TGF-β1 or the bleomycin group. Raltegravir ameliorated pulmonary fibrosis by reducing the pathology score, collagen deposition, and expression of α-smooth muscle actin, NLRP3, HMGB1, TLR4, inhibitor of kappa B, p-NF-κB, HIF-1α, collagen I, and collagen III. The results of this study demonstrate that RAV attenuated experimental attenuates pulmonary fibrosis by inhibiting NLRP3 activation.

## Introduction

Idiopathic pulmonary fibrosis (IPF) is an irreversible, chronic, progressive, and life-threatening disease with limited treatments and poor prognosis. The disease accelerates during acute IPF exacerbation (Hyzy et al., 2007; Lopez et al., 2009; Richeldi et al., 2011; Margaritopoulos et al., 2012). To date, the pathogenesis of IPF is not completely understood, and current therapies are limited to those that reduce the rate of functional decline in patients with mild to moderate disease (Mora et al., 2017).

The most common symptom of IPF is dyspnea. Hypoxia induces a phenotypic switch of fibroblasts to myofibroblasts (Misra et al., 2010). In a hypoxic environment, fibroblasts are activated, which result in abnormal and exaggerated extracellular matrix deposition that mainly contains fibrillary collagen, leading to lung scarring and architectural distortion (Talmadge et al., 2011). In turn, more severe hypoxia occurs, leading to the exacerbation of IPF. Hypoxia-inducible factor-1α (HIF-1α) is a heterodimeric transcription factor that exerts pivotal roles in inducing cellular responses to hypoxia throughout the lung in an O_2_ concentration-dependent and time-dependent manner. HIF-1α overexpression results in the proliferation and transformation of fibroblasts (Mora et al., 2017; Song et al., 2018). Targeting HIF-1α has been suggested as a novel therapy for fibrosis (Xiong and Liu, 2017).

Raltegravir, an inhibitor of the human immunodeficiency virus-1 (HIV-1) integrase, has been used to treat HIV/acquired immunodeficiency syndrome. Raltegravir decreases in the degree of hepatic steatosis in HIV-infected patients with nonalcoholic fatty liver disease (Macías et al., 2017). Nonalcoholic fatty liver disease is accompanied by liver fibrosis, suggesting that RAV may have therapeutic potential for fibrosis. In addition, HIV-1 infection induces NLRP3 inflammasome activation and elevated plasma high mobility group box 1 (HMGB1) levels (Trøseid et al., 2010; Guo et al., 2014). Raltegravir prevents the HIV protease inhibitor-induced inflammatory response and foam cell formation by inhibiting endoplasmic reticulum (ER) stress (Cao et al., 2010). The nucleotide-binding oligomerization domain-like receptor 3 (NLRP3) inflammasome and HMGB1/toll-like receptor 4 (TLR4) play vital roles in inflammation and pulmonary fibrosis, and NLRP3 is a key regulator of HMGB1 release (Hamada et al., 2008; Willingham et al., 2009; Volkova et al., 2012; Stout-Delgado et al., 2016; Wang et al., 2019). The expression of HMGB1 is elevated in the later stages of acute exacerbation in IPF (Ebina et al., 2011). To date, no study has investigated whether RAV attenuates experimental pulmonary fibrosis. Thus, we explored the effects of RAV on experimental pulmonary fibrosis *in vivo* and *in vitro*, and a novel pharmacological target is proposed.

## Materials and Methods

### Chemicals

Raltegravir (purity > 98%; no. 518048-05-0) and MCC950 (purity > 99%, a NLRP3 inhibitor; no. 210826-40-7) were purchased from Hanxiang Biomedical Company (Shanghai, China). Rabbit polyclonal antibodies against NLRP3, HMGB1, TLR4, phosphorylated nuclear factor-κB (p-NF-κB), prolyl hydroxylase domain protein 2 (PHD2), alpha smooth muscle actin (α-SMA), collagens I and III, and mouse polyclonal antibodies against inhibitor of kappa B (IκBα) and HIF-1α were purchased from Abcam Biotechnology (Shanghai, China). BAY11-7082 and TAK-242 were purchased from Beyotime Company (Shanghai, China).

### Cell Culture

Mouse lung fibroblasts (L929) cells were obtained from the Cell Bank of the Chinese Academy of Sciences (Beijing, China). The cells were maintained in MEM containing 10% newborn calf serum, 100 U/ml penicillin, and 100 μg/ml streptomycin at 37°C under a humidified at atmosphere of 5% CO_2_ and 95% N_2_. These were subcultured at an initial density of 1 × 10^5^/ml every 3–4 days.

### Bleomycin-Induced Pulmonary Fibrosis Model

Fifty adult male Sprague–Dawley rats (200–220 g, body weight) were housed in a standard animal laboratory at consistent temperature (22°C ± 2°C) and humidity (60 ± 10%), with free access to chow and water. After 7 days of adaptation, an animal model was established as previously described (Qu et al., 2015). Briefly, the animals were anesthetized, and a pulmonary fibrosis model was induced by single intratracheal instillation of 6 mg/kg bleomycin (BLM) (Invitrogen, Carlsbad, CA, USA) in 0.2 ml saline. The control rats received an equal amount of saline. Twenty-six rats of the BLM model were randomly divided into two groups based on body weight on day 21: BLM model and RAV group. The RAV group was intragastrically administered RAV 80 mg/kg/day, and the control and model groups were intragastrically administered equal amounts of sodium carboxymethyl cellulose. On day 35, lung tissues were collected, and lung coefficients were determined using the following equation: lung coefficient = wet lung weight/body weight × 100%. Then, the tissues were divided into two parts: the left lungs were fixed in 4% paraformaldehyde for histology, and the right lungs were placed in liquid nitrogen for Western blot analysis.

Before starting the main experiment, an exploratory preliminary experiment was performed. The preliminary experiment used five groups, with 10 rats in each group, which included sham, BLM, BLM plus RAV 40 mg/kg/day, 80 mg/kg/day (dosage selection based on its daily dosage 400 mg, BID, in AIDS and preexperiment of anti-BLM-induced fibrosis in rats), or 160 mg/kg/day, respectively. The method and duration of administration were the same as those of the main experiment. Indexes such as body weight, lung coefficient, and hydroxyproline (Hyp) levels were monitored.

### Histopathological Analysis

The middle 1/3 of the left lung tissue was collected and fixed in 4% paraformaldehyde for 48 h, dehydrated, paraffin embedded, and sliced into sections of 4.5-μm thickness. The tissue sections were placed onto a polylysine-coated slide and then immersed in xylene for deparaffinization, and then the sections were rehydrated across an alcohol gradient and stained with hematoxylin and eosin. To measure lung fibrosis, each field was individually assessed for the degree of interstitial fibrosis and graded using a scale of 0 to 8 as follows: grade 0, normal lung; grade 1, isolated alveolar septa with subtle fibrotic changes; grade 2, fibrotic changes of alveolar septa with knot-like formation; grade 3, contiguous fibrotic walls of alveolar septa; grade 4, single fibrotic masses; grade 5, confluent fibrotic masses; grade 6, large contiguous fibrotic masses; grade 7, air bubbles; and grade 8, fibrous obliteration and separately scored in a blinded manner. Masson’s trichrome was used to measure collagen deposition, and the percentage of pulmonary fibrosis was assessed as previously described (Ashcroft et al., 1988; Wang et al., 2002; Lopez et al., 2009).

### Measurement of Hyp Levels

Lung samples were washed with saline and then hydrolyzed with 6 ml/L hydrochloric acid at 100°C for 5 h. Hyp levels were assessed using p-dimethylaminobenzaldehyde at 560 nm and expressed as mg/g wet lung tissue.

### Western Blot Analysis *In Vivo*

Right lung tissues were homogenized with radioimmunoprecipitation assay (RIPA) buffer containing protein inhibitors, after which the supernatant was collected, and the supernatant concentration was measured using the BCA method. Total protein (50 μg) was resolved by 8%–10% sodium dodecyl sulfate-polyacrylamide gel electrophoresis (SDS-PAGE), and protein expression was analyzed with specific antibodies against NLRP3, HMGB1, TLR4, IκBα, p-NF-κB, HIF-1α, α-SMA, collagens I and III, and β-actin (1:1,000). Band destinies were scanned and quantified with ImageJ software and normalized to that of β-actin.

### Immunohistochemical Staining *In Vivo*

The expression of SMA in the lungs was examined using immunohistochemistry (Sun et al., 2018). Tissue sections (4-μm thickness) were deparaffinized and rehydrated, and then treated in 0.01 M citric acid at 400 W in a microwave for 10 min. Endogenous peroxidase was inactivated with 5% H_2_O_2_ in methanol for 30 min at room temperature in the dark. Next, the sections were sealed with a serum cap for 30 min and incubated with rabbit polyclonal anti-α-SMA for 16 h at 4°C, and then washed and incubated with anti-rabbit horseradish peroxidase-conjugated antibody for 60 min at 37°C. The samples were observed under a light microscope, and optical densities were analyzed.

### Analysis of L929 Proliferation

To assess the proliferation of L929 cells, 2 × 10^3^ cells were inoculated into 96-well plates and cultured overnight. The medium was removed followed by the addition of medium alone (control) or medium containing varying concentrations of RAV (3, 10, and 30 μM) with or without transforming growth factor beta 1 (TGF-β1) (10 ng/ml; Sigma, St. Louis, MO, USA) and incubated for 72 h. To further verify the mechanism of cell proliferation, the cells were treated with TGF-β1 for 72 h with or without 5 μM MCC950 (an NLRP3 inhibitor), 1 μM of BAY 11-7082 (an NF-κB inhibitor), and 1 μM of TAK-242 (a TLR4 antagonist). Cell proliferation was assessed using a BeyoClick^™^ 5-ethynyl-2′-deoxyuridine (EdU) cell proliferation kit with TMB, which is based on EdU as a novel alternative for the BrdU (5-bromo-2’-deoxyuridine) assay to directly measure active DNA synthesis or S-phase synthesis of the cell cycle through reaction with fluorescent azides in a Cu(I)-catalyzed [3 + 2] cycloaddition. The absorbance was measured at 630 nm and calculated as a ratio against untreated cells.

### Evaluation of Protein Expression in TGF-β1-Stimulated L929 Cells

Mouse lung fibroblasts (L929) cells were cultured in MEM containing 10% (v/v) fetal bovine serum (FBS) in a 5% CO_2_ and 95% N_2_ humidified atmosphere at 37°C. The cells were grown to about 60% confluency and treated with 10 μM RAV with or without TGF-β1 (10 ng/ml) for 72 h. Then, the protein expression levels of NLRP3, HMGB1, TLR4, p-NF-κB, PHD2, HIF-1α, α-SMA, and collagens I and III were assessed by Western blotting. To investigate the possible mechanism of lung fibrosis, the cells were treated with TGF-β1 (10 ng/ml) for 72 h with or without MCC950 (5 μM), BAY11-7082 (1 μM) or TAK-242 (1 μM), and NLRP3, TLR4, p-NF-κB, and HMGB1 expressions were evaluated by Western blotting.

### Immunoblot Analysis *In Vitro*

L929 cells were cultured for 72 h and washed thrice with ice-cold phosphate-buffered saline. Then, the cells were lysed with RIPA buffer containing a protein enzyme inhibitor [phenylmethylsulfonyl fluoride (PMSF)]. The cell supernatant was collected, proteins were extracted, and protein concentrations were determined using the BCA assay. Proteins (30 μg) were separated by 8%–10% SDS-PAGE and blotted onto nitrocellulose membranes (Bio-Rad, Hercules, CA, USA) to detect the protein expression levels according to a previous method (Yang et al., 2018). Proteins were analyzed by Western blotting with specific antibodies against NLRP3, HMGB1, TLR4, p-NF-κB, PHD2, HIF-1α, and collagens I and III, using β-actin as loading control. The results are expressed as fold increase relative to the control group. Protein bands were quantified with Image J software.

### Statistical Analysis

The Wilcoxon rank-sum test was used to analyze the different groups of lung tissues. A one-way analysis of variance (ANOVA) followed by a Dunnett’s test was used to assess differences in statistical significance. Quantitative data were expressed as the mean ± standard deviation. Statistical significance was set at P < 0.05.

## Results

### Effects of RAV on Lung Coefficients and Histopathological Changes in Lung Tissues

**Figure 1:**
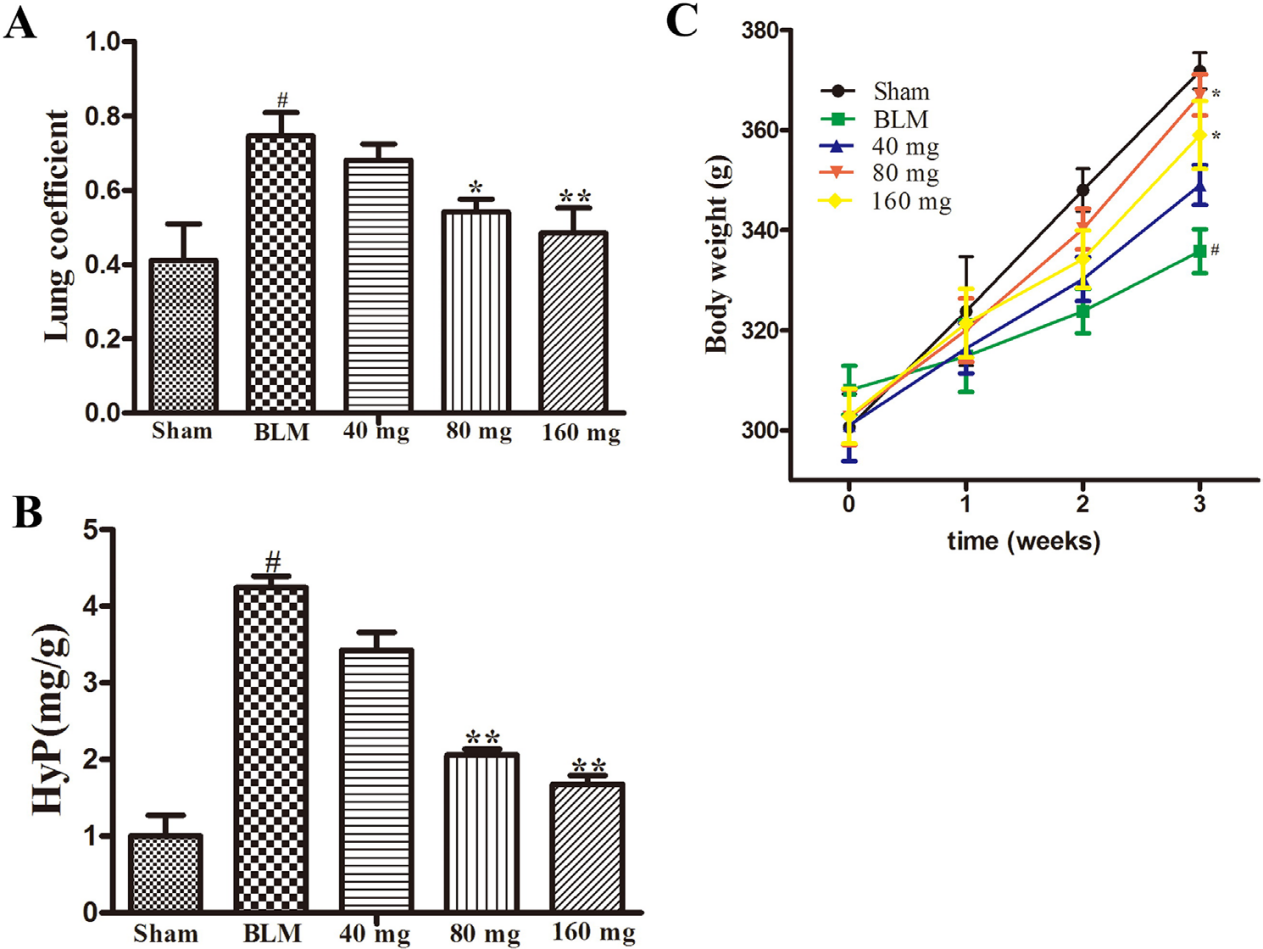
Effects of RAV on the lung coefficient (Part A), Hyp (Part B) and body weight (Part C) in a preliminary experiment. All data are presented as the mean ± SD (n = 8). ^#^p < 0.01 vs. the sham group; *p < 0.05, **p < 0.01 vs. the BLM group. Notable difference was ascertained by ANOVA along with Dunnett’s test.

The results of the preliminary experiment showed that the application of RAV at doses of 40 mg/kg/day, 80 mg/kg/day, and 160 mg/kg/day resulted in an increase in body weight, by reduced lung coefficients and Hyp levels (**Figure 1**). Compared with 80 mg/kg, the rats in the 160 mg/kg group had lower body weight gain, and we selected the dose of 80 mg/kg for the subsequent investigation on the effects of RAV on experimental pulmonary fibrosis *in vivo*.

**Figure 2:**
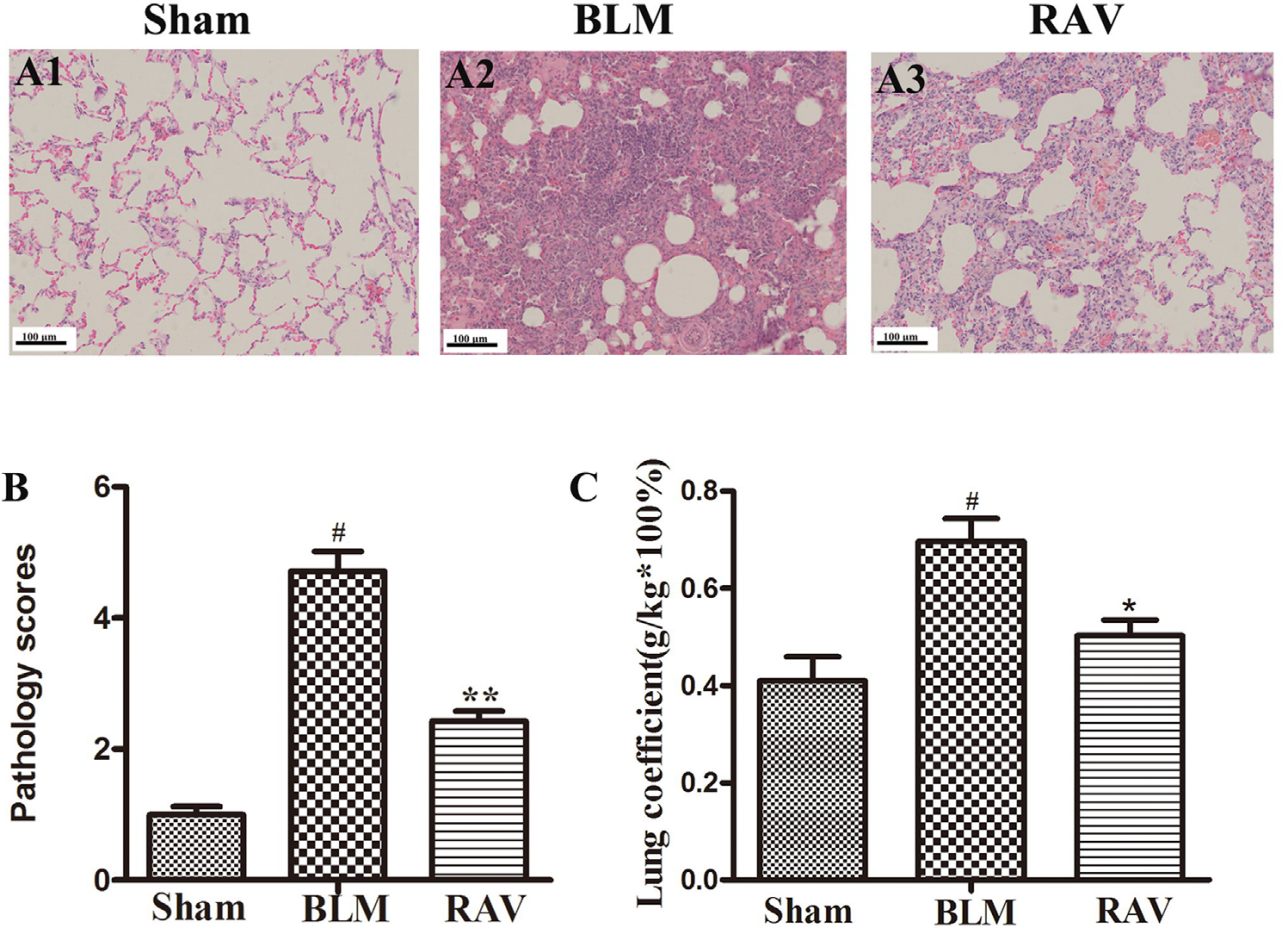
Effects of RAV on lung coefficients and histopathological changes in lung tissues. Representative images of hematoxylin and eosin (H&E) staining **(A1–A3)**. Effects of RAV on the histopathological score **(B)**. Effects of RAV on lung coefficients in vivo after RAV and BLM treatment **(C)**. All data are presented as the mean ± SD (n = 8). ^#^p < 0.01 vs. the sham group; *p < 0.05, **p < 0.01 vs. the BLM group. Notable differences were ascertained using ANOVA along with Dunnett’s test.

Lung tissues were semiquantitatively assessed, and we found no inflammatory or fibrotic changes in the normal tissue (**Figure 2A1**). In addition, the pathology score in RAV-treated rats significantly decreased relative to the BLM-induced rats (**Figures 2A2, A3**). Pulmonary coefficient is a measure of pulmonary fibrosis (Song et al., 2018). During pulmonary fibrosis, the lung coefficient significantly increases. The weight of the rats was recorded before the rats were sacrificed, and the weight of the lung tissues was recorded when the lung was isolated from the donor mice, which was used to calculate the lung coefficient. The lung coefficient of the control and RAV-treated groups decreased compared to the BLM model group (**Figure 2C**).

### Effects of RAV on Collagen Levels in Lung Tissues

**Figure 3:**
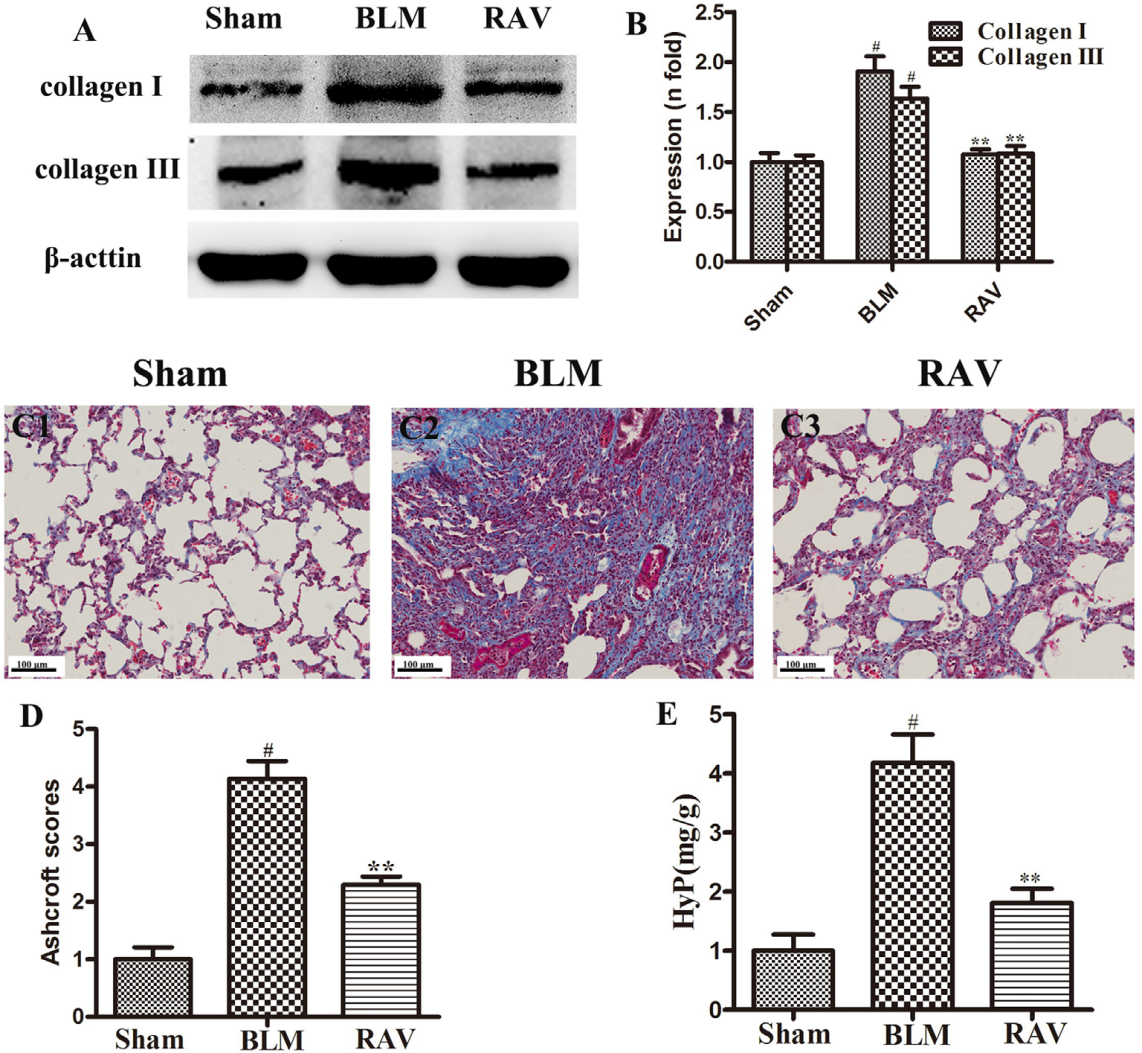
Effects of RAV on collagen levels in lung tissues. Representative images of Masson’s staining **(C1–C3)**. Effects of RAV on collagen content by western blot analyze **(B)**, Aschcroft scores **(D)** and Hyp **(E)** in rats after RAV and BLM treatment. All data are presented as the mean ± SD (n = 8). ^#^p < 0.01 vs. the sham group; *p < 0.05, **p < 0.01 vs. the BLM group. Notable differences were ascertained by ANOVA along with Dunnett’s test.

To further observe BLM-induced changes, collagen content was examined by Western blotting and Masson’s trichrome staining. The protein expressions of collagens I and III were measured by Western blotting. On day 35, collagens I and III expression increased in BLM animals relative to the sham group (p < 0.01). Nevertheless, collagens I and III expression in the RAV-treated group decreased compared to the model group (p < 0.05; **Figures 3A, B**). Compared to the sham group, the model group had a large amount of collagen deposition in the lung tissue (**Figures 3C1, C2**). However, collagen content significantly decreased in the RAV-treated rats relative to the BLM-induced rats (**Figures 3C3, 3D**). In addition, Hyp content as another index of collagen accumulation was examined to assess lung fibrosis. Collagen content in the RAV-treated group was significantly lower than that of the BLM model group (**Figure 3E**). In addition, Hyp content significantly decreased in the RAV-treated animals relative to the model animals.

### Effects of RAV on Protein Expression *In Vivo*

**Figure 4:**
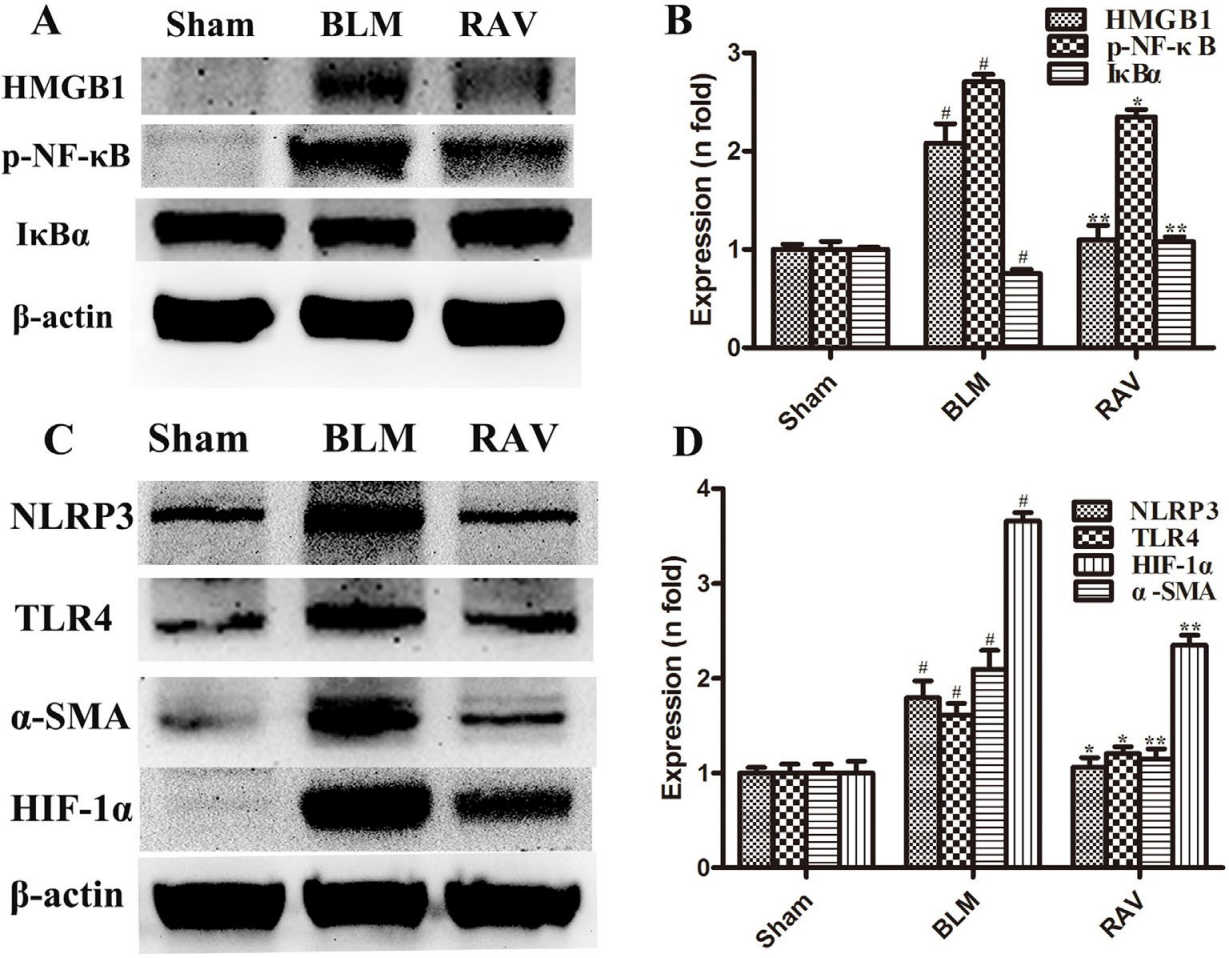
Effects of RAV on protein expression levels *in vivo*. HMGB1, p-NF-κB and IκBα **(A** and **B)**, NLRP3, TLR4, α-SMA and HIF-1α **(C** and **D)** expression levels were analyzed. All data are presented as the mean ± SD (n = 8). ^#^p < 0.01 vs. the sham group; *p < 0.05, **p < 0.01 vs. the BLM group. Notable differences were ascertained by ANOVA along with Dunnett’s test.

**Figure 5:**
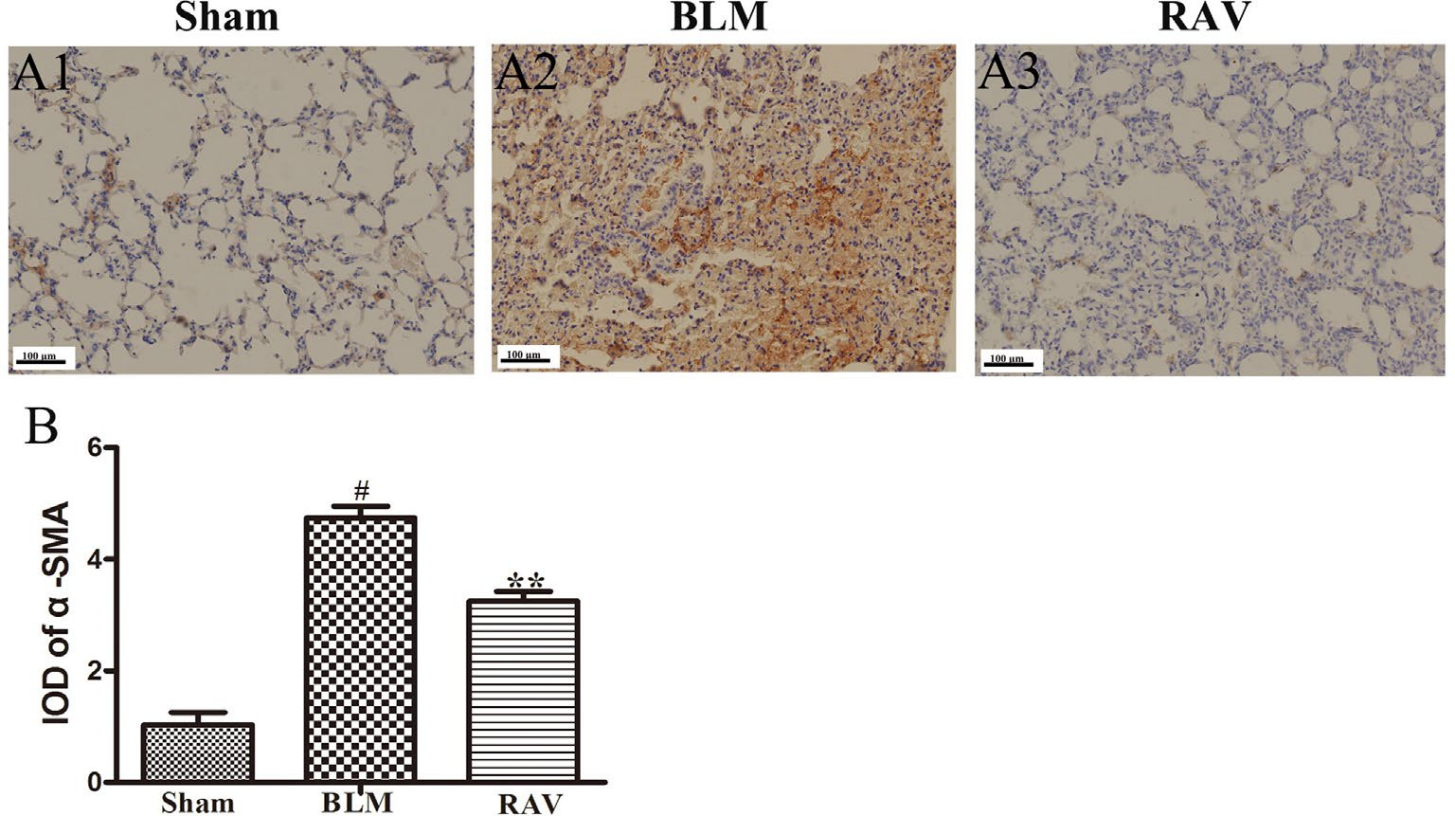
Effects of RAV on changes in α-SMA by immunohistochemical staining of lung tissues. The representative images of immunohistochemical staining **(A)** and IDO of α-SMA **(B)**. All data are presented as the mean ± SD (n = 8). ^#^p < 0.01 vs. the sham group; **p < 0.01 vs. the BLM group. Notable differences were ascertained by ANOVA along with Dunnett’s test.

The protein expression of NLRP3, HMGB1, TLR4, IκBα, p-NF-κB, HIF-1α, and α-SMA was measured by Western blotting. In addition, immunohistochemical staining of lung fibroblast marker, α-SMA, was evaluated in the lung tissues. On day 35, all protein expression increased relative to the sham group, except for IκBα, which decreased (p < 0.05 or p < 0.01). Nevertheless, protein expression in the RAV-treated group decreased compared to the model group, except for IκBα, which increased (p < 0.05 or p < 0.01; **Figure 4**). The results of Western blotting of α-SMA coincided with that of immunohistochemical staining. Compared to the sham animals, α-SMA expression increased in the BLM-treated animal, but decreased in the RAV-treated animals (**Figures 4** and **5**; p < 0.01).

### Effects of RAV on L929 Cell Proliferation

**Figure 6:**
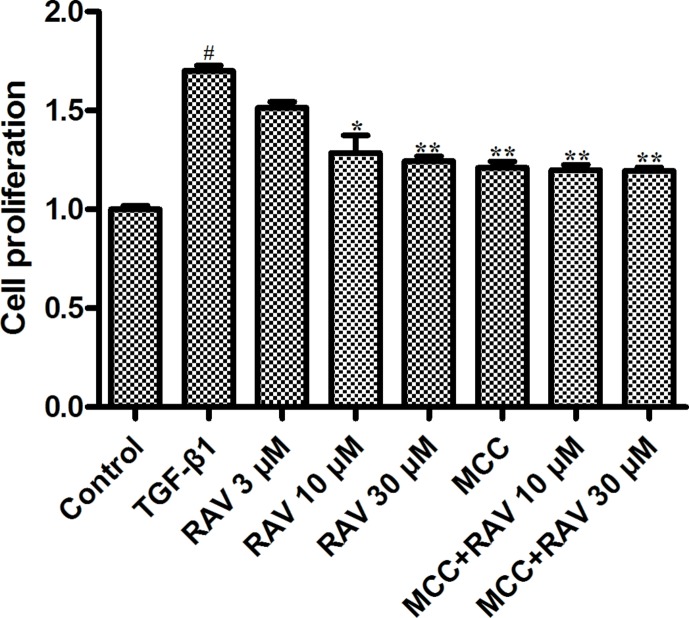
Effects of RAV on L929 cell proliferation. Cells proliferation was tested by EdU cell proliferation kit with TMB after TGF-β1-induced L929 cells 72 h. All data are presented as the mean ± SD (n = 3). ^#^p < 0.01 vs. the control group; *p < 0.05, **p < 0.01 vs. the TGF-β1 group. Notable differences were ascertained by ANOVA along with Dunnett’s test.

TGF-β1 (10 ng/ml) was used to stimulate L929 cells to mimic the profibrotic environment (Pardo et al., 2005). The TGF-β1-treated cells showed significantly higher proliferation rates compared to the control group. The L929 cells were treated with RAV (3, 10, 30 μM), and cell proliferation rates were significantly inhibited compared to the TGF-β1 group. To further study the mechanisms underlying cell proliferation, the L929 cells were treated with MCC950 (5 μM) or MCC950 (5 μM) plus RAV (10, 30 μM). The results showed that cell proliferation was significantly inhibited in both the MCC950 and MCC950 plus RAV groups. Cell proliferation in these groups did not further decrease (**Figure 6**). These findings show that RAV attenuates L929 cell proliferation by inhibiting NLRP3 activation.

### Effects of RAV on L929 Cell Proliferation and Protein Expression *In Vitro*

**Figure 7:**
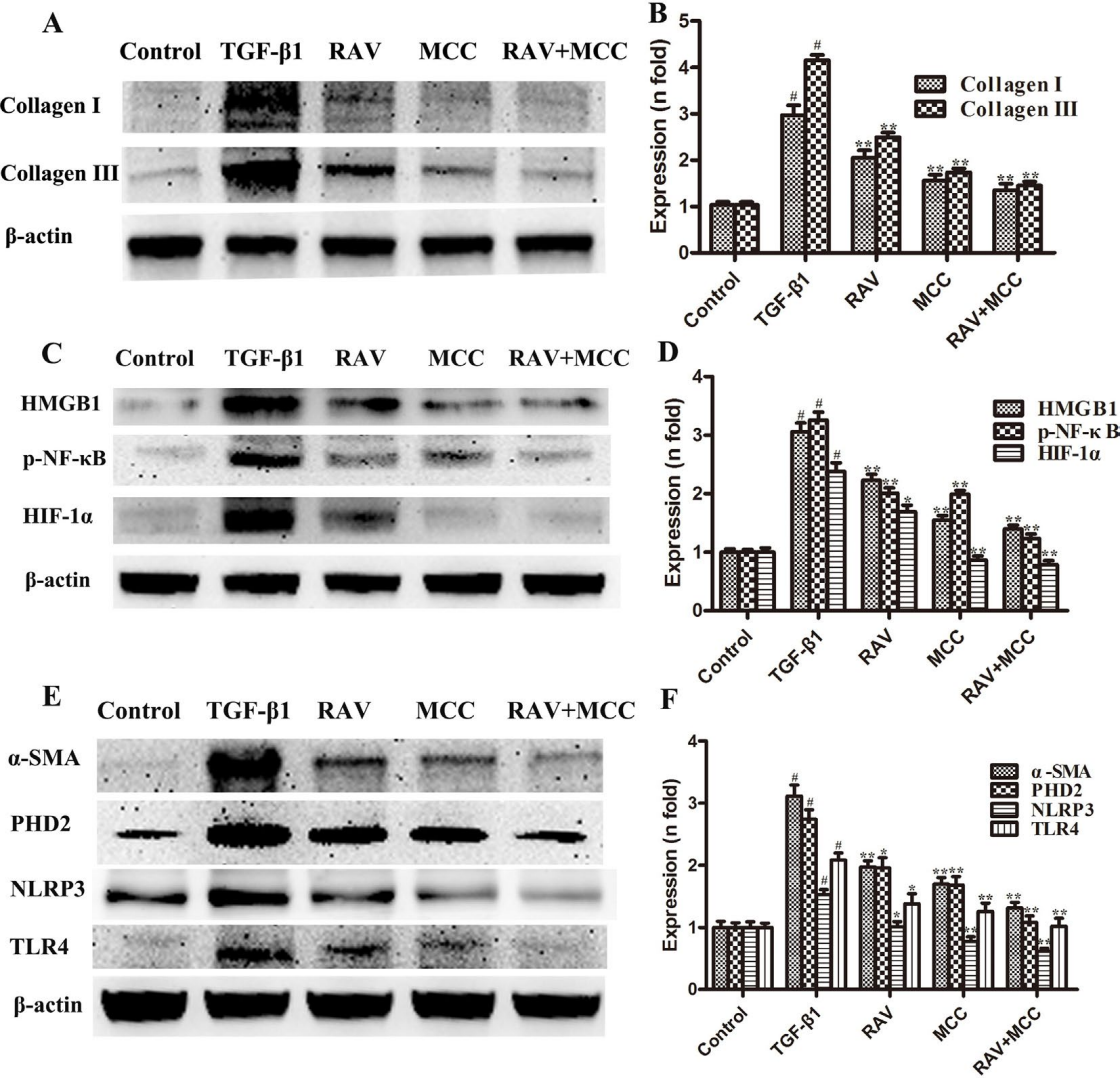
Effects of RAV on changes in protein expression levels with NLRP3 activation *in vitro*. After TGF-β1-induced L929 cells for 72 h, the expression levels of proteins were examined by Western blotting after RAV, TGF-β1, and MCC950 (MCC) treatment. Collagen I and Collagen III **(A** and **B)**, HMGB1, p-NF-κB and HIF-1α **(C** and **D)**, α-SMA, PHD2, NLRP3 and TLR4 **(E** and **F)** expression levels were analyzed. All data are presented as the mean ± SD (n = 3). All data are presented as the mean ± SD (n = 3). ^#^p < 0.01 vs. the control group; *p < 0.05, **p < 0.01 vs. the TGF-β1 group. Notable differences were ascertained by ANOVA along with Dunnett’s test.

**Figure 8:**
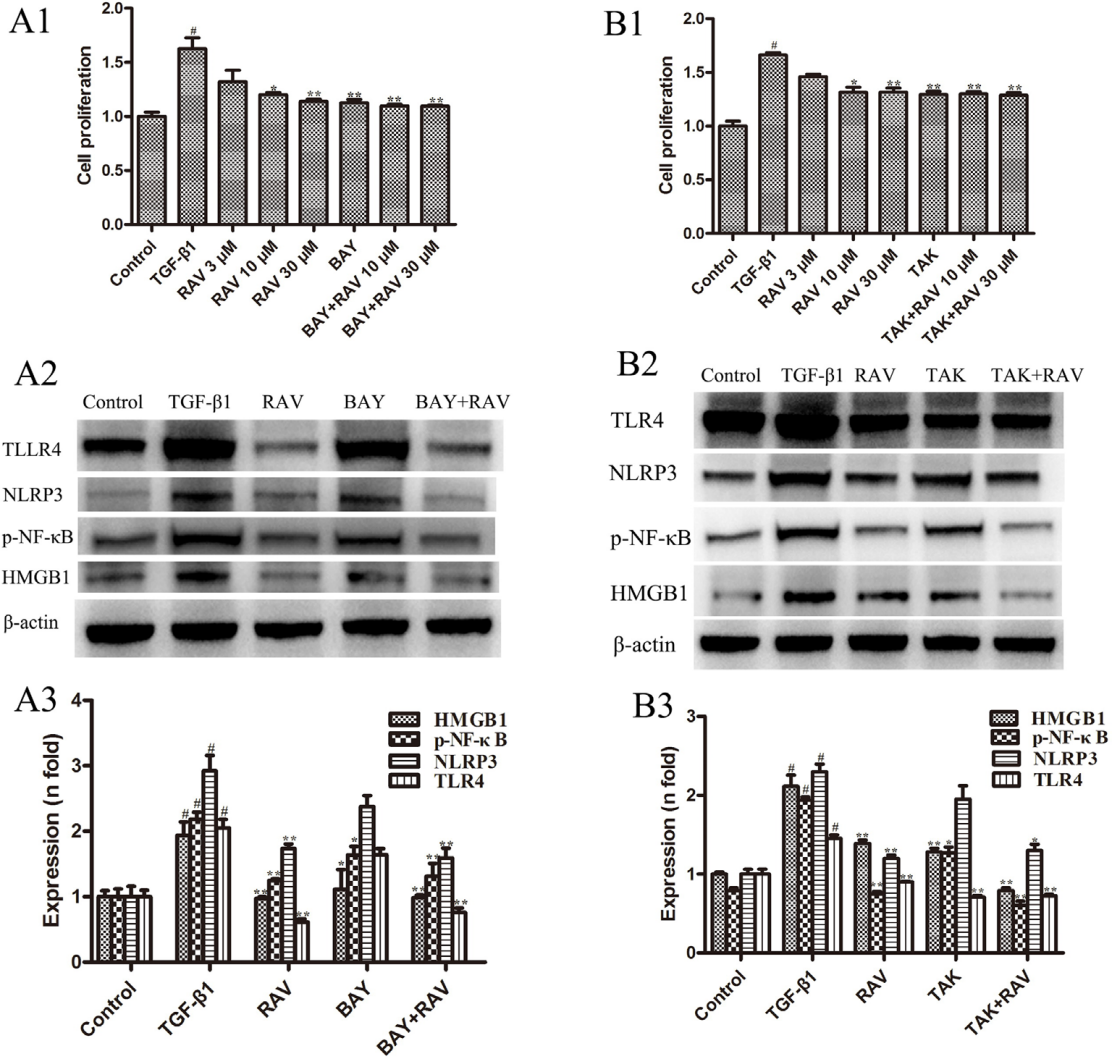
Effects of RAV on changes in HMGB1/TLR4 and p-NF-κB expression *in vitro*. After TGF-β1-induced L929 cells for 72 h, L929 proliferation was analyzed by EdU cell proliferation kit with TMB **(A1** and **B1)**, and the expressions of NLRP3, TLR4, HMGB1 and p-NF-κB with or without BAY **(A2** and **A3)**, with or without TAK **(B2** and **B3)** were examined by Western blotting. All data are presented as the mean ± SD (n = 3). ^#^p < 0.01 vs. the control group; *p < 0.05, **p < 0.01 vs. the TGF-β1 group. Notable differences were ascertained by ANOVA along with Dunnett’s test.

The L929 cells were cultured in MEM to approximately 60% confluency. Then, the cells were stimulated with TGF-β1 (10 ng/ml) and treated with RAV (10 μM) for 72 h. Then, L929 cell proliferation was analyzed and the proteins were extracted and examined, as shown in **Figures 6**–**8**. Compared to the control group, protein expression of collagens I and III significantly increased after the cells were stimulated with TGF-β1, but their expression was significantly inhibited with RAV treatment compared to the control. After TGF-β1-induced profibrosis, the protein expressions of TLR4, HMGB1, PHD2, HIF-1α, and p-NF-κB significantly increased compared to the control, but decreased following RAV treatment in comparison to the TGF-β1-stimulated group. NLRP3 protein expression increased with TGF-β1 treatment, but decreased with RAV treatment.

To determine the mechanism underlying NLRP3 activation, MCC950 was used to silence NLRP3 activation. Then, L929 cell proliferation was analyzed. The protein expression levels of NLRP3, TLR4, HMGB1, PHD2, HIF-1α, p-NF-κB, collagens I and III were examined after incubation with RAV (10 μM) with or without 5 μM MCC950 with TGF-β1 for 72 h. The results showed that all protein expression and L929 cell proliferation with and without MCC950 decreased compared to the TGF-β1 treatment group. In addition, MCC950 alone reduced the expression of proteins and the extent of cell proliferation, whereas MCC950 plus RAV did not further reduce protein expression levels or cell proliferation rates (P > 0.05; **Figures 6** and **7**). These findings show that RAV attenuates experimental lung fibrosis by inhibiting NLRP3 activation.

To determine whether the mechanism of decreasing lung fibrosis was caused by NF-κB activation, an NF-κB inhibitor, BAY11-7082, was used to silence NF-κB activation. Then, L929 cell proliferation was analyzed. The protein expression levels of NLRP3, TLR4, HMGB1, and p-NF-κB were examined after incubation with RAV (10 μM) with or without BAY11-7082 1 μM with TGF-β1 for 72 h. The results showed that BAY11-7082 alone reduced p-NF-κB and HMGB1 expression and inhibited cell proliferation, but did not reduce NLRP3 and TLR4 expression (**Figures 8A1, A2**). In contrast, BAY11-7082 plus RAV did not further inhibit cell proliferation or reduce p-NF-κB or HMGB1 expression (p > 0.05).

To determine the mechanism underlying the decrease in lung fibrosis caused by HMGB1/TLR4, a TLR4 antagonist TAK-242 was used to silence HMGB1/TLR4. Then, L929 cell proliferation was analyzed. NLRP3, TLR4, HMGB1, and p-NF-κB expression levels were examined after incubation with RAV (10 μM) with or without TAK-242 1 μM with TGF-β1 for 72 h. The results showed that TAK-242 alone reduced TLR4, HMGB1, and p-NF-κB expression and inhibited cell proliferation, whereas it did not reduce NLRP3 expression (p > 0.05; **Figures 8B1, B2**). These findings show that RAV attenuates experimental lung fibrosis by inhibiting NLRP3/HMGB1/TLR4 signaling.

## Discussion

IPF is an irreversible, progressive, and fatal disease with a poor prognosis. A number of treatments have been investigated in the past. However, the clinical treatment option of IPF is limited. Many of the therapeutic strategies reduce fibrosis in preclinical models, but are ineffective or even potentially harmful in humans, which include interferon-γ (IFNγ), the TNF receptor inhibitor etanercept, the endothelin receptor antagonist bosentan and macitentan, the phosphodiesterase 5 inhibitor sildenafil, and the chemotherapeutic drug imatinib mesylate. Only nintedanib and pirfenidone have been approved by the Food and Drug Administration (FDA) (Mora et al., 2017) to improve lung function. Thus, it is essential to identify novel therapeutic targets for IPF treatment. In this study, the effects of RAV on experimental pulmonary fibrosis and its associated mechanisms *in vitro* and *in vivo* were investigated. Importantly, this is the first study to report the effect of RAV in pulmonary fibrosis in a preclinical model and explore its potential in the clinical treatment of IPF.

TGF-β1 is probably the most potent profibrotic factor, and downregulation of the TGF-β pathway is an attractive target for IPF therapy, including GC1008, GSK3008348, and BG00011, which are currently undergoing clinical trials (Mora et al., 2017). TGF-β1 activation promotes the proliferation and differentiation of fibroblasts into myoblasts, which results in collagen production and intra-alveolar fibrosis (Zhang et al., 2015). Thus TGF-β1 was utilized to stimulate L929 cells and mimic the proliferative environment *in vitro*. During pulmonary fibrosis, pulmonary fibroblast proliferation plays a vital role. A marked pathological feature of pulmonary fibrosis is hypoxia, which contributes to fibrotic diseases (Senavirathna et al., 2018). Nuclear HIF-1α protein is involved in many pathophysiological processes under hypoxia (Zhu and Zhao, 2017). PHD2, an enzyme mostly responsible for oxygen-induced degradation of HIF-α protein, plays a major role in IPF (Song et al., 2018). In addition, HMGB1 plays an important role in fibroblast proliferation (Hamada et al., 2008). Our study found that the proliferation of L929 cells with RAV treatment was inhibited compared to the TGF-β1 treatment group, and HMGB1, PHD2, and HIF-1α expression levels significantly decreased with RAV treatment relative to TGF-β1-induced cells and BLM-induced rats.

IPF is characterized by fibroblast proliferation and the abnormal accumulation of extracellular matrix (ECM) molecules, particularly fibrillar collagens. Compared to natural pulmonary tissue-induced fibroblasts, lung fibrosis-induced fibroblasts and myfibroblasts secrete more ECM, primarily collagen types I and III (Bocchino et al., 2010; Baum and Duffy, 2011), so the content of collagen in lung tissues can directly reflect the degree of pulmonary fibrosis in animals. The extent of collagen deposition is reflected by the amount of Hyp content (Tanaka et al., 2009), and collagen deposition in local tissues can reflect the severity of pathology by collagen staining to evaluate the degree of antifibrosis of drugs or tested compounds.

In fibroblasts, HMGB1 significantly increases collagen deposition (Lee et al., 2018) and plays an important role in pulmonary fibrosis (Hamada et al., 2008). It directly stimulates fibroblast proliferation, participates in fibrogenesis (Wang et al., 2013), and increases α-SMA expression (Lynch et al., 2010). In addition, HMGB1 induces fibrosis by binding to the TLR4 receptor and activating NF-κB nuclear translocation (Wang et al., 2013). A marker of myofibroblasts, α-SMA, has higher expression levels in patients with pulmonary fibrosis and affects the survival rate of patients (Ramos et al., 2001; Qu et al., 2015). In addition, plasma HMGB1 levels are elevated in HIV-1-infected patients and reduced with effective antiretroviral therapy (Trøseid et al., 2010). NLRP3 inflammasome activation results in collagen deposition (Wree et al., 2014). NLRP3 is associated with IPF in both animal and human samples (Terlizzi et al., 2018). NLRP3 activation and TLR4/NF-κB pathway are closely related (Ahn et al., 2015; Jo et al., 2016). Our studies have shown that HMGB1, Hyp, and collagen levels all increased in the BLM rat model. However, HMGB1, Hyp, and collagen levels all decreased in RAV-treated rats. In addition, the lower expression levels of α-SMA, HMGB1, TLR4, NLRP3, and p-NF-κB were noted in RAV-treated animals and TGF-β1-stimulated L929 cells.

Drug repositioning has many merits, particularly in reducing the risk and cost of research and development. Raltegravir was approved for medical use in the United States in 2007 as an antiretroviral medication that is used together with other medications for the treatment of HIV/AIDS. In addition, RAV is generally well tolerated when used in combination with optimized background-therapy regimens in treatment-experienced patients with HIV-1 infection in trials of up to 48 weeks duration (Croxtall and Keam, 2009), so no additional tolerance testing is required. Low risk of treatment with RAV will be conducted in evaluation of IPF therapy for long time.

## Conclusions

In summary, the results of this study demonstrated that RAV inhibits cell proliferation *in vitro* and reduces the expression of NLRP3, HMGB1, TLR4, PHD2, p-NF-κB, HIF-1, α-SMA, and collagens I and III, whereas increases the expression of IκBα *in vitro* and *in vivo*. Therefore, attenuation of experimental pulmonary fibrosis might be inhibited by NLRP3 activation and highlight its potential use as a novel multitarget drug for IPF.

## Data Availability

The raw data supporting the conclusions of this manuscript will be made available by the authors, without undue reservation, to any qualified researcher.

## Ethics Statement

This study was conducted in accordance with the recommendations of the Institutional Animal Care guidelines and the National Institutes of Health *Guide for the Care and Use of Laboratory Animals* (Bethesda, MD, USA). The protocol was approved by the Committee on the Ethics of Animal Experiments of Binzhou Medical University (Permit No. SCXK 20170003).

## Author Contributions

XZ performed the research, analyzed the data, and wrote the manuscript. HH and GZ contributed to animal experiments. DL and HW revised the manuscript. WJ designed and funded the research, interpreted the data, and finally approved the submission of this manuscript.

## Funding

The study was supported by project ZR2019MH045, Shandong Provincial Natural Science Foundation, China and the Dominant Disciplines’ Talent Team Development Scheme of Higher Education of Shandong Province.

## Conflict of Interest Statement

The authors declare that the research was conducted in the absence of any commercial or financial relationships that could be construed as a potential conflict of interest.
